# Obstacle negotiation in older adults: prefrontal activation interpreted through conceptual models of brain aging

**DOI:** 10.1093/geroni/igaa057.1584

**Published:** 2020-12-16

**Authors:** David Clark, Sudeshna Chatterjee, Rachael Seidler, Jared Skinner, Dorian Rose, Paige Lysne, Adam Woods

**Affiliations:** 1 University of Florida, Gainesville, Florida, United States; 2 Applied Physiology and Kinesiology, Gainesville, Florida, United States; 3 Appalachian State University, Boone, North Carolina, United States; 4 Aging and Geriatric Research, Gainesville, Florida, United States; 5 Clinical and Health Psychology, Gainesville, Florida, United States

## Abstract

Age-related decline in executive function is associated with walking deficits in older adults. The main objective of this study was to better understand the cognitive control of obstacle negotiation in older adults by identifying predictors of prefrontal recruitment during the task. The study also examined the association between prefrontal recruitment and walking performance as well as interpretation of prefrontal activity relative to cognitive models of brain aging. Prefrontal oxygenated hemoglobin concentration (O2Hb) was measured by functional near-infrared spectroscopy during typical walking (Typical) and obstacle negotiation (Obstacles) tasks in older adults. The primary outcome was change in prefrontal recruitment (∆PFR), measured as Obstacles ∆O2Hb minus Typical ∆O2Hb. Stepwise regression was used to identify potential predictors of ∆PFR. Additional analyses were conducted to further examine the relationship between ΔPFR and the identified predictors. Greater ∆PFR was predicted by lower age, worse executive function, and their interaction (R2=0.19 , p=0.02). Particularly, the effect of executive function on ∆PFR was more pronounced for “early aging” than for “late aging” older adults (p<0.001). Greater ∆PFR was significantly associated with a smaller reduction in walking speed during Obstacles compared to Typical. In conclusion, age, executive function, and their interaction predict prefrontal recruitment during obstacle negotiation in older adults. These findings are generally consistent with existing cognitive models of brain aging including neural inefficiency, compensatory overactivation, and capacity-limitation with a recruitment ceiling effect.

